# Young «oil site» of the Uzon Caldera as a habitat for unique microbial life

**DOI:** 10.1186/s12866-020-02012-1

**Published:** 2020-11-24

**Authors:** Sergey E. Peltek, Alla V. Bryanskaya, Yuliya E. Uvarova, Aleksey S. Rozanov, Timofey V. Ivanisenko, Vladimir A. Ivanisenko, Elena V. Lazareva, Olga V. Saik, Vadim M. Efimov, Sergey M. Zhmodik, Oxana P. Taran, Nikolay M. Slynko, Sergey V. Shekhovtsov, Valentin N. Parmon, Nikolay L. Dobretsov, Nikolay A. Kolchanov

**Affiliations:** 1 Laboratory of Molecular Biotechnologies of Federal research center Institute of Cytology and Genetics of the Siberian Branch of the Russian Academy of Sciences, 10 Lavrentiev Aven., Novosibirsk, Russia 630090; 2 Kurchatov Genomics Center of Federal research center Institute of Cytology and Genetics of the Siberian Branch of the Russian Academy of Sciences, 10 Lavrentiev Aven., Novosibirsk, Russia 630090; 3 Novosibirsk State University, Pirogova str., 2, Novosibirsk, Russia 630090; 4 The V.S. Sobolev Institute of Geology and Mineralogy SB RAS, pr. Koptyuga, 3, Novosibirsk, Russia 630090; 5 Institute of Chemistry and Chemical Technology SB RAS, FRC Krasnoyarsk Science Center SB RAS, Akademgorodok, 50/24, Krasnoyarsk, Russia 660036; 6 Siberian Federal University, Svobodny ave. 79, Krasnoyarsk, Russia 660041; 7 Boreskov Institute of Catalysis SB RAS, pr. Lavrentieva 5, Novosibirsk, Russia 630090; 8 Trofimuk Institute of Petroleum Geology and Geophysics SB RAS, pr. Koptyuga, 3, Novosibirsk, Russia 630090

**Keywords:** Oil site, Microbial communities, Metabolic pathways, The Uzon Caldera

## Abstract

**Background:**

The Uzon Caldera is one of the places on our planet with unique geological, ecological, and microbiological characteristics. Uzon oil is the youngest on Earth. Uzon oil has unique composition, with low proportion of heavy fractions and relatively high content of saturated hydrocarbons. Microbial communities of the «oil site» have a diverse composition and live at high temperatures (up to 97 °C), significant oscillations of Eh and pH, and high content of sulfur, sulfides, arsenic, antimony, and mercury in water and rocks.

**Results:**

The study analyzed the composition, structure and unique genetics characteristics of the microbial communities of the oil site, analyzed the metabolic pathways in the communities. Metabolic pathways of hydrocarbon degradation by microorganisms have been found. The study found statistically significant relationships between geochemical parameters, taxonomic composition and the completeness of metabolic pathways. It was demonstrated that geochemical parameters determine the structure and metabolic potential of microbial communities.

**Conclusions:**

There were statistically significant relationships between geochemical parameters, taxonomic composition, and the completeness of metabolic pathways. It was demonstrated that geochemical parameters define the structure and metabolic potential of microbial communities. Metabolic pathways of hydrocarbon oxidation was found to prevail in the studied communities, which corroborates the hypothesis on abiogenic synthesis of Uzon hydrothermal petroleum.

**Supplementary information:**

**Supplementary information** accompanies this paper at 10.1186/s12866-020-02012-1.

## Background

The caldera of the Uzon Volcano (Kamchatka Peninsula, Russia) is a region with active hydrothermal activity, which contains outlets of unique natural hydrothermal petroleum [1, 2]. Hydrothermal petroleum is the oil found in natural outlets within active hydrothermal fields [3–5]. According to C^14^ dating, hydrothermal petroleum from various regions of the Earth is modern, with the oldest sample being 29,000 years old [1]. The Uzon petroleum is the youngest on Earth, with initial estimates at 1000 years [6], and later found to be only 50 years old [7].

The composition of the Uzon oil was investigated in several studies [6, 8–12]. It belongs to the methane–naphtene–aromatic type [10], i.e., heavy low-tar petroleum with the prevalence of hydrocarbons. There is twice as more saturated hydrocarbons (57–58%) than aromatic ones (30–32%), with only 10–13% of tar and asphaltene components, and less than 0.3% of asphaltens [12].

There are two main hypotheses on the origin of hydrothermal petroleum: (1) abiogenic synthesis by the Fischer–Tropsch process; (2) conversion of organic sediments and/or microorganisms [13–16]. A comprehensive study of hydrothermal petroleum as the youngest product of naphtide genesis, analysis of composition, metabolism, and ecology of microbial communities inhabiting oil sites will ultimately reveal the mechanisms of oil formation. In our earlier studies, geology and geochemistry of the main oil site of the Uzon Caldera were examind [2]. This paper continues this interdisciplinary research and investigates microbial communities of the oil site using metagenomics.

There are only three known hydrothermal petroleum sites in island arcs: the Ngawa [17] and Waiotapu [18] in New Zealand, the Wakamiko Caldera in Japan [19], and the Uzon Caldera in Kamchatka, Russia. The Rainbow and the Calcite Springs of the Yellowstone Park (USA) located in a caldera of supervolcano created by a large mantle plume also have outlets of hydrothermal petroleum [4]. Although the geological settings of Uzon and Yellowstone differ, their surface thermal outlets are similar both in morphology and geochemical properties.

The Uzon Caldera has high surface temperature, which is similar to that found at 2–3 km depth in classic oil and gas basins [7]. In these conditions, organic matter from the Pliocene and low Quaternary sediments is converted to oil hydrocarbons and heterocyclic compounds. It is noteworthy that the forming petroleum inherits the hydrocarbon skeleton and lipid stereochemistry of the source organics. These young formations have more biomarkers than normal oils.

The aim of this work was to determine the composition and structure of microbial communities of the Uzon oil site using 16S rRNA sequencing, to estimate the potential of microbial communities for hydrocarbon degradation, as well as to search for the relations between taxonomic composition, completeness of metabolic pathways (the quantity and nature of enzymatic reactions), and geochemical parameters.

### The Uzon Caldera as the habitat of microbial communities

The Uzon Geyser depression is located in the middle of the East Kamchatka Volcanic Belt, between the Kikhpinych and Taunshitz volcanoes [2]. Several stages of caldera formation within the 50–270 ky interval are known [20–23].

Currently the Uzon Caldera harbors multiple thermal outlets with water temperatures raging from 30 to 100 °C that are found within five large thermal fields, as well as numerous smaller springs, formed by three main directions of faults [2]. The composition and physicochemical parameters are diverse [2, 22, 24, 25].

The oil site is the major petroleum seep located on the periphery of the section III of the Eastern Thermal Field, which is probably the axial region of the Uzon Caldera hydrothermal system. The size of it is 40 × 15 m and it harbors two natural thermal outlets, a small unnamed outlet in the center of the field, and the Yashcheritsa cauldron [2].

The majority of the oil site is covered by rounded pumice (sediments of a paleolake); the western part, closer to the Yashcheritsa spring, by gray muddy clay with predominance of kaolinite. Within the field, water temperatures in sampling pits (manually dug pits that are soon field with groundwater) ranges from 9 to 90 °C. Oscillations of Eh and pH are also high. Almost all sampling pits have oil film or droplets on the water surface, with the most oil concentrating in the northern part of the Field. In the southern part, minerals of the main ore body can be found at the depth of 20–30 cm. The ore body is located in section II of the Eastern Thermal Field and consists of realgar, auripigment, antimonite, pyrite, cinnabar, and metacinnabarite [22, 24–26].

### The composition of the Uzon oil

Chemical analysis of the Uzon oil was the subject of two studies [11, 12]. The Uzon oil predominantly consists of hydrocarbons (90–93%), mostly of saturated ones (twice as much as of aromatic hydrocarbons) [11]. The concentration of heterocyclic compounds is 7–10% (here and henceforth % denote mass %), while that of asphaltens is less than 0.3% [27, 28].

The composition of saturated and aromatic hydrocarbons suggests that they were formed by lipids from sedimented aboveground plants: the C_29_ / C_27_ steranes ratio is over 2.5, tricyclic index (2 (C_19_ + C_20_) / ΣC_i_, i = 23, 24, 25, 26) exceeds 2.0, relatively high concentrations of triaromatic steroids, aquatic life of the caldera and bottom waters (n-C_27_ / n-C_17_ < 0.2, Pr / Ph < 0.5) and living matter of bacteria (gopans) is observed. Biogenic origin of lipid is also confirmed by the carbon isotopes composition of fossilized organic matter (δ^13^C = − 28.3 ‰). Judging by hydrocarbon biomarkers, the degree of transformation of the initial organic matter corresponds to the very beginning of the main phase of oil formation, as suggested by the ratio of even and odd n-alkanes (close to 1), high concentrations of steranes with the absence of biohopanes above C_27_, prevalence of the S isomers over the R isomers in homobiphenes, low values of hopane Ts/Tm ratio, and low phenanthrene and dibenzothiophene indices (0.6–1.1 and 0.3–1.2, respectively) [11].

### Microbial communities of the Uzon oil site

The Uzon Caldera, a unique natural phenomenon, was studied by many prominent scientists: B.I. Piip, S.I. Naboko, G.F. Pilipenko, N.G. Beskrovnyi, G.A. Zavazin, and others [29]. Various collectives of researchers studied the microflora of thermal fields of the Uzon Caldera, the Geyser valley, and other thermal outlets of the Kuril-Kamchatka Volcanic Belt [30–32].

The microflora of the Uzon oil site was also previously studied [33, 34]. Depending on the period of study and the methodological base, these microbial communities were investigated using methods of classical microbiology, molecular biology, genomics, proteomics, and bioinformatics [35]. These studies yielded a large amount of data on microorganisms of Kamchtka as a whole, as well of the oil site in particular [36–38].

A group of researchers led by E. A. Bonch-Osmolovskaya studied thermophilic organisms from the Geyser valley and the Uzon Caldera [36]. They found that such groups as Actinobacteria, Bacteroidetes, Aquificales, Deinococcus-Thermus, Thermodesulfobacteria, Verrucomicrobia, Actinobacteria, Bacteroidetes, Firmicutes, etc., were prevalent in these communities.

Metagenomics was used to study genetic diversity of microbial communities in several outlets of the Uzon Caldera with different temperature and pH. For example, Gumerov [39] analyzed taxonomic diversity of the following natural and artificial outlets of the Uzon Caldera: the Zavarzin and Burlyashchiy springs, as well as points 1884, 1810, 1805, and 1807. The Point 1884 and the Zavarzin spring are located near the Uzon oil site.

The Point 1884 was a sampling pit where hydrocarbons were carried to the surface by thermal waters. It was probably located near the oil site (author’s comment). Over 70% of this microbial community was represented by archaeal sequences, 90% of them uncultured. Gumerov [39] suggest that the Point 1884 also harbors yet unidentified lithoautotrophs, or else it depends on the influx of organics with rain and/or deep layers with geothermal currents.

The Burlyashchiy spring located near the Uzon oil site was also studied in detail [39, 40]. According to 16S pyrosequencing, lithoautotrophs (Aquificales and Thermoproteales) prevailed in its water, while in the sediments they were found together with Crenarchaeota, Korarchaeota, and bacteria of the genus *Caldimicrobium* (Thermodesulfobacteria) [40].

Since the Point 1884 and the Burlyashchiy spring are hot and contain hydrothermal petroleum, their microbial communities might utilize certain oil components, such as high molecular weight n-alkanes, cycloalkanes, polycyclic aromatic hydrocarbons, terpenes, pristane, phytane, and other resilient compounds, and/or survive in habitats with high concentration of these substances [33]. They might even contain enzymatic pathways capable of yet unknown biochemical transformations of oil hydrocarbons.

Dobretsov et al. [2] determined archaeal diversity in three samples from the Eastern Thermal Field of the Uzon Caldera, i.e., the Yashcheritsa spring and two sampling pits. Slutskaya et al. [41] discovered new groups of uncultured prokaryotes with unique biochemical properties in microbial communities of Kamchatka. Samples from hot springs with low pH yield promising strains capable to degrade oil. Metagenomic data on natural microbial communities could provide us with new microorganisms for soil bioremediation and biotechnologies of oil procession, as well as to shed light on the mechanisms of oil formation.

## Results

### Geochemistry of the oil site water

Comparison of geochemical characteristics of water of the oil site and other parts of the Uzon Caldera was already examined earlier [2, 22]. Thermal waters of the Uzon Caldera (including those of thermal springs, mud and water cauldrons, mud volcanos, etc.) formed one area and three trends (Additional file 1: Fig. S1). Our calculations demonstrated that they correspond to stability boundaries of various forms of sulfur and iron [2].

Almost all pore solutions from the sample pits lied on the trend II that corresponds to the stability lines of H_2_S and FeS_2_. Some were close to the trend IV that most probably reflected that stability boundary of Fe^2+^ and its oxidation to Fe^3+^, which causes the formation of iron oxides and hydroxides. Only the water from the Yashcheritsa spring belonged to trend III describing the oxidation of HSO_3_^−^ to S_2_O_6_^2−^, or the most complex transition of sulfur from the thiosulfate to the polythionate form. The Burlyashchiy spring that lies at a distance from the oil site, belonged to area I (Additional file 1: Fig. S1).

The sampled locations differed in temperature, pH, and Eh (Additional file 2: Table S1). Sulfate and chloride ions dominated in the majority of samples, except for the Burlyashchiy spring that harbors a considerable part of hydrocarobonate ions. Sampling pits with high oil content had more chlorides compared to sulfates, which may indicate significant influx of thermal waters. Sodium was the prevalent cation in all solutions; only the U3–2-3 sample had a significant amount of calcium, as well as the highest detected mineralization (1.3 g/l).

Like all studied waters of the Uzon Caldera, the sampled points had high content of alkaline, alkaline earth, and anionogenic elements. Samples with low pH contained high amounts of iron and aluminium group metals, as well as rare metals (Additional file 3: Fig. S2).

### Structure of microbial communities

A total of 332,576 sequences at least 250 bp long were obtained (Table 1) that grouped into 1414 clusters each corresponding to a definite taxon. Most of the representative sequences for clusters had over 95% sequence similarity to 16S rRNA of known microorganisms.

**Table 1:** Number of sequences and operational taxonomic units (OTUs) in the studied samples

Sample	Initial number of reads (sequences)	Number of reads before quality control (excluding chimeric ones)	Number of reads longer than 250 bp	% of reads longer than 250 bp	OTUs
U3_kot	33,436	26,305	15,875	60.34974	55
U3_AS (U-003-3-3/11)	29,664	23,117	11,899	51.47294	106
U3_1–3	37,597	31,475	21,396	67.97776	102
U3_2–3	29,404	25,506	13,675	53.61484	177
U3_4–9	35,763	28,885	19,721	68.27419	206
U3_4–10	44,969	35,903	29,101	81.05451	150
U3_5yasher	41,817	34,346	26,071	75.90695	130
U20Bur	30,409	21,406	11,795	55.10137	204
U5–1	23,066	22,509	21,689	94.03017	106
G11-1а	26,451	25,392	22,480	84.98734	178

Current studies of microbial communities indeed use more reads. However,the number of reads obtained in our study allow us to detect the presenceof species that account for up to 0.1–0.01% of the number of individuals,and to study metabolic pathways within the communities.

The number of the obtained sequences, as well of the obtained reads after quality control, ranged from 53 to 94%. Variation in OTU number was higher, from 55 in a small natural outlet «Kotelok» to 206 in the U3_4–9 sampling pit and 204 in the Burlyashchiy spring; it is worth noting that the number of OTUs did not correlate with the number of reads.

**Fig. 1:**
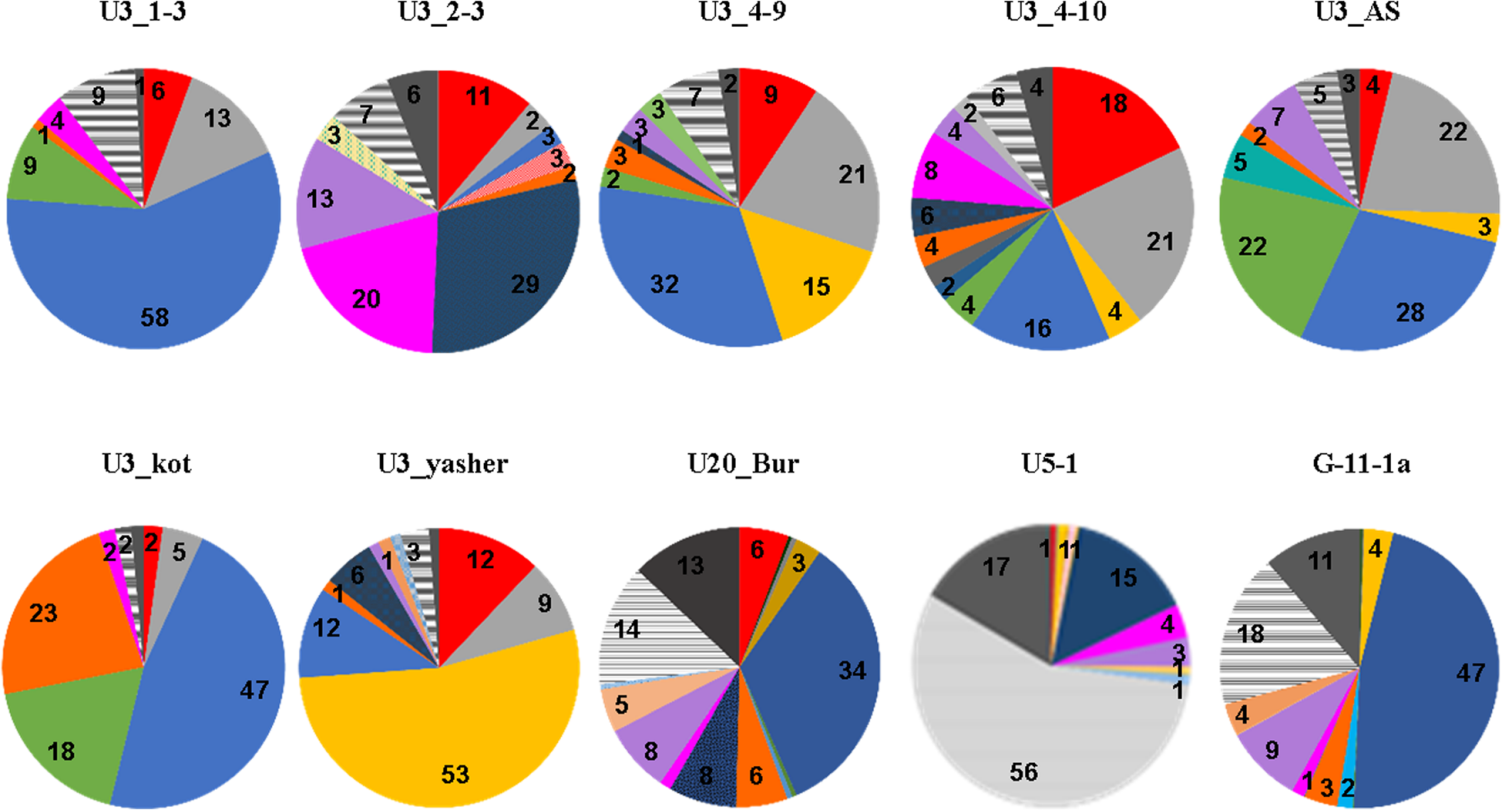
Dominant prokaryote groups in the studied communities

The detailed information on the structure of the studied microbial communities is given in Additional file 4: Table S2, Additional file 5: Table S3, Additional file 6: Table S4. Bacteria were detected in all samples, while archaea (Crenarchaeota) were found only in natural outlets: U3–5yashcher, U20Bur, and U5–1. *Sphingomonas* (Alphaproteobacteriа), Betaproteobacteria, *Pseudomonas* (Gammaproteobacteria), Deltaproteobacteria, Actinobacteria, Bacillales, Lactobacillales, Bacteroidetes, and Cyanobacteria were abundant in all or almost all sampling points, while Proteobacteriа/Gammaproteobacteria (*Acinetobacter*)*,* Firmicutes/Halanaerobiales, Firmicutes; Thermoanaerobacterales, Aquificae, Fusobacteria*,* Thermotogae were rare and were found only in one or two samples (Fig.1, Additional file 7: Fig. S3).

The maximum number of OTUs (32) was found in the U3_2–3 sample and belonged to the most abundant group of bacteria across all samples -Bacteroidetes type. The majority of the rest of the taxa was represented by few OTUs. The highest microbial diversity was found in the natural outlets UBur and G11-1a; the lowest, in the U3_1–3 sampling pit (Table 2).

**Table 2:** Diversity indices

Sample	Shannon index	Inverse Simpson index
U3-AS	3.5	16.13
U3–1-3	2.3	3.42
U3–2-3	3.9	20.01
U3–4-9	3.9	22.59
U3–4-10	3.7	17.17
U3–0-kot	2.8	11.48
U3–5-yasch	2.9	7.68
U20-Bur	4.1	28.99
U5–1	2.9	5.39
G11-1а	3.9	26.01

## Discussion

The majority of the studied sites contained representatives of Actinomycetales (Actinobacteria)*.* Actinomycetales are soil aerobic bacteria capable of degrading resilient substrates [42] that have acid-resistance cell walls, which enable them to survive at low pH. For example, representatives of the genus *Rhodococcus* that was also found in the studied sites are long known to inhabit surfaces containing petroleum [43, 44]. These bacteria can exist in a wide range of temperatures and pH, and their lipophilic cell wall allows them to passively absorb petroleum hydrocarbons. The genus is characterized by the presence of several genes of the alkB alkane dehydrogenase family with different substrate specificity, which allows its representatives to live on a wide range of organic substrates [45, 46]. However, certain species have high substrate specificity. For example, *Rhodococcus rhodochrous* KUCC 8801 from Kuwait can rapidly grow on linear alkanes with 12 to 20 carbon atoms, but is unable to utilize short and branched alkanes, as well as aromatic hydrocarbons [47]. *Rhodococcus salmonicolor* oxidized side chains of aromatic hydrocarbons, as well as terminal methyl groups of n-alkanes to carboxylic groups with subsequent β-oxidation [48]. *Rhodococcus sp.* BPM 1613 can utilize various isoprenoid hydrocarbons, i.e., pristane, phytane, and farnesan. After the oxidation of the terminal methyl group, pristane can undergo either β-oxidation, or ω-oxidation to dicarboxylic acids [49]. Rhodococci, like other alkane-degrading microorganism, can produce emulsifiers [50], that increase the surface of hydrocarbons in water and thus the rate of decomposition.

Up to 21.7% of sequences in certain sampling points belonged to the genus *Pseudomonas*, which includes mesophilic and obligate aerobic chemoorganotrophic bacteria. Some strains of that genus are capable of metabolizing hydrocarbons [51, 52]. The alkB alkane monooxygenase is the catalyst of the alkane dehydrogenase complex that provides the first stage of alkane degradation. The alkB operon was found, e.g., in the OCT plasmid of *Р. putida* GPo1 that is amenable to horizontal transfer [53]. *Pseudomonas* is known to decompose alkanes to alcohols and carboxylic acids.

Enterobacteriaceae (Gammaproteobacteria) was another abundant group in the studied samples. This group is immensely diverse in morphology and metabolic capacities [54]. Gammaproteobacteria MFB021 strain (Enterobacteriaceae) was isolated from petroleum-contaminated soil from India and was found to degrade oil components at salinity level reaching 25% [55].

Bacteria of the genus *Bacillus* (Bacillales), detected in the majority of the studied samples, are known to decompose oil hydrocarbons at high temperatures. For example, *Bacillus thermoleovorans* strains B23 and H41 from Japan can grow at 50–80 °C, with the optimum of 70 °C for B23 and 65 °C for H41 [56]. These strains efficiently (up to 60%) degrade n-alkanes longer than C_12_ and C_15_, respectively. Triglycerides of the cell wall of both strains were found to contain palmitate and stearate when grown on n-heptadecane, which suggests that they oxidize methyl groups of n-alkanes with subsequent β-oxidation. Eleven strains of the genus *Geobacillus* (Bacillales) grown on a mixture of C_10_-C_20_ saturated hydrocarbons contained a set of three to six alkB genes, only two of which were common for all studied strains [57].

All sampling points, except for the control G11-1a, harbored Alphaproteobacteria*.* The genus *Sphingomonas* belonging to this class contains Gram-negative rod-shaped chemoheterotrophic obligate aerobes having catechol 1,2-dioxygenase (1-pyrocatechase, 1.13.11.1) that has an important role in decomposition of aromatic substances [58]. These bacteria can grow on organic substrates at low temperatures; e.g., the Ant 17 strain from contaminated soil of the Scott Base in the Antarctica survives multiple freeze-thaw cycles and is capable to grow on and metabolize substrates containing aromatic fragments at 1 to 35 °C [59].

*Burkholderia* (Betaproteobacteria) is a genus of Gram-negative mobile obligate aerobic rod-shaped bacteria. *Burkholderia* sp. are able to degrade a wide range of natural pollutants [60]. Different components of oil pollutants were shown to be processed at different rates: a bacterial consortium consisting mostly of *Burkholderia* sp. decreased the amount of hexadecane 12-fold (by 60% by the 21st day), and of pristane, twice as much as of phenanthrene [61]. Representatives of the genus *Methylobacterium* (Alphaproteobacteria) are obligate aerobes, mesophiles, and chemoorganotrophes. Their ability to degrade components of the vaseline oil, as well as aromatic substances and medium- and short-chain alkanes was also described [62].

There is no data on the role of other microorganisms found by us in primary oxidation of carbohydrate components of oil. However, they have the potential to act as the energy-storage part of the microbial community by metabolizing the resulting alcohols and carbohydrates. Moreover, minor components are known to have a crucial impact in metabolic pathways of a community [63]. For example, representatives of Hydrogenothermaceae (Aquificae) are typical extremophiles living in hot springs, sulfur baths, as well as thermal outlets of the ocean floor. These bacteria were earlier found in warm waters of the Hengill volcano (Iceland) [64]. Other rare groups were Thermotogae*,* Gram-negative anaerobic thermophiles capable of metabolizing complex hydrocarbons and producing hydrogen [65], as well as Thermodesulfobacteria, sulfate reducing thermophiles that have an important role in primary uptake of minerals. *Thermoanaerobacter uzonensis* initially described by Wagner was also detected [66]. It is a heterotrophic Gram-positive spore-forming thermophilic anaerobe isolated from hot springs of Uzon. Its temperature optimum lies at 61°С, and the pH, at 7.1, with the growth range of pH 4.2 to 8.9 [67].

### Completeness of the KEGG metabolic pathways in the Uzon oil site microbial communities

In order to test the hypothesis whether microbial communities are able to degrade the volcanogenic oil, as hypothesized in [2, 7, 11], the completeness of metabolic pathways was analyzed.

It was found that the following metabolic pathways had the highest completeness (over 90%): Synthesis and degradation of ketone bodies, Furfural degradation, D-Glutamine and D-glutamate metabolism, D-Alanine metabolism, Flagellar assembly, Cell cycle – Caulobacter, Xylene degradation, Bacterial chemotaxis, Bacterial secretion system, Phosphotransferase system (PTS), Nitrotoluene degradation, Dioxin degradation, Valine, leucine and isoleucine biosynthesis, Lipopolysaccharide biosynthesis, Benzoate degradation, Styrene degradation, Peptidoglycan biosynthesis. The following ones are involved in hydrocarbon metabolism: Xylene degradation, Nitrotoluene degradation, Dioxin degradation, Benzoate degradation, Styrene degradation. The completeness of the Naphthalene degradation and Benzoate degradation pathways for the U3.4.10 sample is given in Additional file 8: Fig. S4-A, Additional file 8: Fig. S4-B.

It is obvious (Additional file 8: Fig. S4-A, Additional file 8: Fig. S4-B) that the Naphthalene degradation pathway is complete, and the Benzoate degradation pathway is mostly complete. This might indicate that the studied Uzon communities are capable of hydrocarbon destruction. Enzymes involved in the Ethylbenzene degradation also participate in decomposition of naphtens. Additional file 9: Fig. S5 shows the completeness of the Ethylbenzene degradation pathway for two samples from the oil site, the U3.4.10 pit characterized by the maximum observed completeness, and the U3kot natural outlet with the lowest completeness. It is noteworthy that U3.4.10 contained many oil drops, while U3kot had no visible oil.

**Fig. 2:**
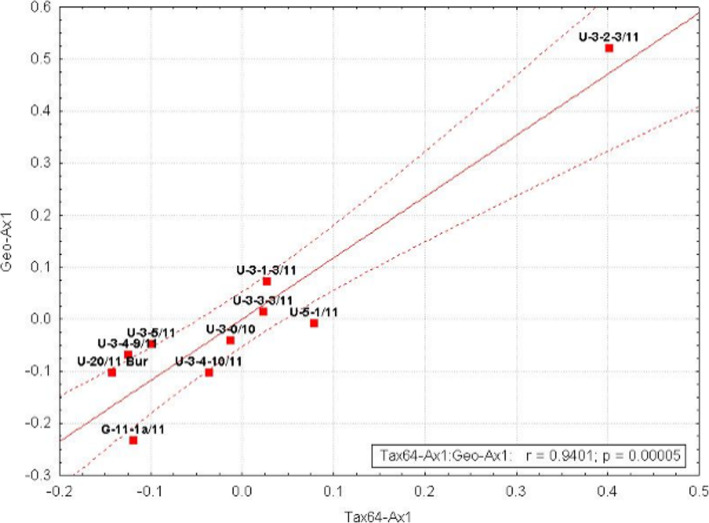
Correlation between taxonomic composition and geochemical parameters. Tax64-Axis 1, the component of microbial diversity; Geo-Axis 1, the component of geochemical parameters; red squares denote the studied samples; dotted red lines, 0.95 confidence intervals

Two-block partial least-square sanalysis demonstrated statistically significant correlation between the first 2B-PLS axis (geochemical parameters: TDS, Na^+^, K^+^, Cl^−^, B, Cu, Ga, Ge, etc.) and taxonomic diversity (0.94 (*p* < 10^− 4^)) (Fig. 2).

Maximum differences for these factors were found for the U3–2-3 sample (рН 2.85, 67 ^о^С), high differences were also found for the G11-1a control sample from the Geyser valley. Other samples had no significant differences in these coordinates.

**Fig. 3:**
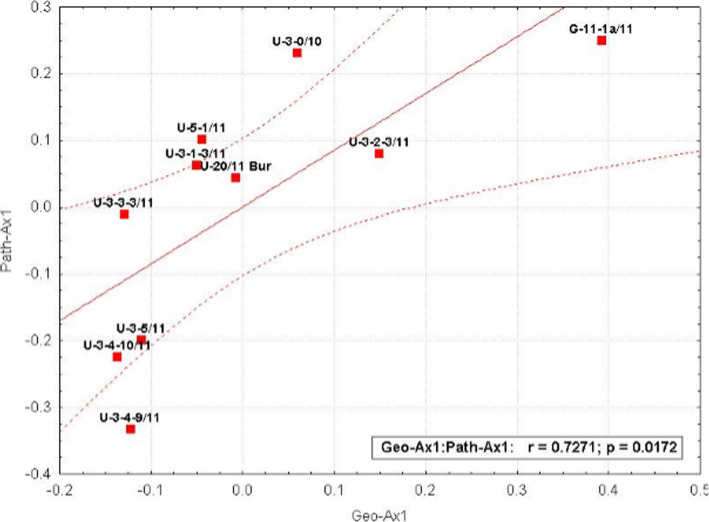
Correlation between completeness of metabolic pathways and geochemical parameters. Path-Axis 1, the component of pathway completeness; Geo-Axis 1, the component of geochemical parameters; red squares denote the studied samples; dotted red lines, 0.95 confidence intervals

The correlation between the primary 2B-PLS axis (geochemical parameters: TDS, Na+, K+, Cl-, B, Cu, Ga, Ge, etc.) and completeness of metabolic pathways was 0.73 (*p* < 0.05) (Fig. 3).

Samples with high oil content (U3–4-9, U3–4-10, U3_AS, the Yashcheritsa spring U3–5) had minimal differences in the geochemical component, as well as the following samples: the Burlyashchiy spring U-20, the Thermophilic spring U5–1, and the U3–1-3 sampling pit. For the component of the completeness of metabolic pathways, the following samples had minimal differences: the Burlyashchiy spring U-20, the Thermophilic spring U5–1, the U3–1-3 and U3–2-3 sampling pits.

The control sample G11-1a demonstrated statistically significant differences for the studied parameters from the rest of the samples, but was similar in the completeness component with the U-3-0 sample (the «Kotelok» natural outlet).

On the whole, in this coordinate system there is a distinct group of samples with high oil content and the Yashcheritsa spring, as well as the Burlyashchiy spring, the Thermophilic spring, and the U3–1-3 sampling pit.

Therefore, our results corroborate the hypothesis that the Uzon microbial communities metabolize oil hydrocarbons of volcanogenic origin.

## Conclusions

The Uzon Caldera is one of the places on our planet with unique geological, ecological, and microbiological characteristics. Although hydrothermal petroleum is found elsewhere, Uzon oil is the youngest on Earth. Our studies across several years (2010–2012) and seasons (May and September) allowed us to describe the Uzon oil site [2].

Microbial communities of the oil site live at high temperatures (up to 97 °C), significant oscillations of Eh and pH, and high content of sulfides, arsenic, antimony, and mercury in water and rocks. Our NGS approach yielded over 300,000 sequences longer than 250 bp, which is similar to that found in other microbial communities of the Uzon Caldera [33, 40] and other geothermal ecosystems [68, 69]. Over 1400 microbial taxa were found. The number of sequences and OTUs was similar in various sample types: natural outlets, sampling pits, and control samples. Bacteria dominated the studied communities, accounting for up to 98.5% of all identified sequences. The majority of the studied sites contained certain microbial taxa that were able not only to survive in extreme environments, but also to metabolize hydrocarbons. Seventeen KEGG metabolic pathways had an average of 90% completeness; five of those were involved in hydrocarbon metabolism.

There were statistically significant relationships between geochemical parameters, taxonomic composition, and the completeness of metabolic pathways. It was demonstrated that geochemical parameters define the structure and metabolic potential of microbial communities. Metabolic pathways of hydrocarbon oxidation were found to prevail in the studied communities, which corroborates the hypothesis on abiogenic synthesis of Uzon hydrothermal petroleum.

## Material and methods

### Object of the study

A series of expeditions to the Uzon Caldera were performed in 2010–2013.

Field analyses. In 2011 and 2012, collected samples of water from the following thermal outlets of the Uzon oil site were collected: the Yashcheritsa, «Kotelok», and Burlyashchiy springs. In addition, pore solutions from manually excavated sample pits along four transects were sampled (Additional file 10: Fig. S6) [2].

In each sampling point, temperature, pH, Eh were measured, water samples for chemical analysis were collected, and the quantity of surface oil was estimated.

Water samples were filtered through 0.45 μm filters; a portion of water was preserved by distilled HNO_3_ (2 ml per 0.5 l of water). Physicochemical parameters (pH, Eh, T) and concentrations of O_2_ and HS^−^ that can significantly change upon storage were determined in the field using an ANION 4151 multichannel combined water analyzer. Water pH was measured using an ESLK-01.7 combined electrode; Eh, by an ERP-101 platinum electrode with an EVL-1 M3.1 silver chloride electrode with reference to the standard electrode potential. For HS^−^ measurements an EKOM-S2^−^ ion selective electrode with an EVL-1 M3.1 silver chloride reference electrode were used. Concentrations of dissolved oxygen were determined using an amperometric sensor of oxygen partial pressure with a gas permeable membrane (Clark electrode). In addition, concentrations of O_2_ and HS^−^ were assayed using MERCK (Germany) mobile tests. Parallel measurements ensured error level below 5–10%.

For metagenomic analysis, a total of eight points in the oil site were sampled, as well as two control points (Table 3). Water with sediments or microbial films was placed in sterile vials and fixed with an equal volume of 96% ethanol.

**Table 3:** Sampling points

Sample name	Sample type
U3_kot	Water with sediments, an unnamed natural outlet in the center of the oil site («Kotelok»)
U3_AS (U-003-3-3/11)	Water with sediments from the sampling pit with high level of arsenic sulphides
U3_1–3	Water with sediments, sampling pit
U3_2–3	Water with sediments, sampling pit
U3_4–9	Water with sediments, sampling pit
U3_4–10	Water with sediments, sampling pit
U3_5yasher	Water with sediments, Yashcheritsa spring
U20Bur	Water with sediments, Burlyashchiy spring
U5–1	Water, Thermophilniy spring
G11-1а	Water, dome formation with multiple outlets (Geyser valley)

A total 30 samples were obtained for the Collection of biotechnological microorganisms as a source of novel promising objects for biotechnology and bioengineering of Federal Research Center “Institute of Cytology and Genetics of the Siberian Branch of the RAS”.

### Laboratory analyses

Elemental composition (Additional file 2: Table S1) in the thermal waters was determined by mass spectrometry with inductively coupled plasma (ICP-MS) on an Agilent Technologies Agilent 7500 quadrupole ICP-MS spectrometer (USA). Analytical accuracy was under 10% if the concentrations were an order of magnitude higher than the detection limit (see Additional file 11: Table S5). Detection limits (see Formula 1) were calculated for each element as 3 × SD of the signal for the control water sample with the concentration of the analyte approaching zero, divided by the sensitivity coefficient (determined by analysis of standard element concentration), as recommended in the application manual of Agilent 7500. Sensitivity was determined by the number of counts per unit concentration.

Formula 1.

$$ \mathrm{DL}=\left(3\sigma \times \mathrm{standard}\ \mathrm{element}\ \mathrm{concentration}\right)/\left(\mathrm{S}-\mathrm{B}\right) $$

where DL is the detection limit; σ, standard deviation (SD) from the control water sample signal; S, standard element signal (concentration 10 μg/l); В, control water sample signal at zero analyte concentration.

### Metagenomic analysis

#### Total DNA extraction

DNA extraction was performed in September 2011. Samples were triturated in a sterile ceramic mortar if necessary. Approximately 300 μl of sample suspension was added to an Eppendorf tube (2 mL). The suspension was centrifuged at 8000×g for 10 min. The pellet was re-suspended by pipetting in 500 μl buffer containing 100mMol Tris-HCl, pH 8, 100mMol EDTA, pH -8.0; 30 μl of chloroform and 200 μl of lysozyme (50 mg/ml) were added, and cells were incubated at 37 °C for 1 h, with shaking at 5 min intervals, and with subsequent addition of 100 μl 10% SDS and 100 μl 10% sarkosyl (Sigma). Three cycles of freezing in liquid nitrogen for 2 min and thawing for 5 min at 65°С were performed. Another freeze-thaw cycle was performed after addition of 100 μl 10% Polyvinylpyrrolidone (Sigma). Then 50 μl of 2.5М СаСl_2_ were added, and the suspension was incubated at 65°С for 10 min with occasional shaking. The supernatant was transferred to a sterile tube, and an equal volume of phenol/chloroform (1/1) was added to the tube. The tubes were vortexed for 2 min and centrifuged at 13,000×g for 10 min. The supernatant (400 μl) was transferred to a new tube containing 100 μl 10 M NH_4_Ac and 1 ml of 96% ethanol, incubated overnight at − 20 °C and centrifuged at 16,000×g for 20 min. The precipitate was washed with 70% ethanol, dried at room temperature and dissolved in water (mQ).

#### Library construction

DNA extracted from the samples was used as a template for amplification of bacterial and archaeal 16S rRNA genes with universal primers: 343F (50-CTCCTACGGRRSGCAGCAG) and 806R (50-GGACTACNVGGGTWTCTAAT) [22, 70]. Reagents for PCR (DMSO, PCR buffer, polymerase, nucleotide triphosphates) were purchased from Agilent Technologies, USA. PCR mix (50 μl) contained 1× herculase buffer, 10 μM of each dNTPs, 10pmoles of forward and reverse primers, 100 ng of DNA, and 2.5u of Herculase. The following amplification profile was used: 3 min at 95 °C; 6 cycles of 15 s at 95 °C, 15 s at 50 °C, and 60 s at 72 °C; 35 cycles of 10 s at 95 °C, 10 s at 55 °C, and 60 s at 72 °C; and an additional elongation phase of 5 min at 72 °C. Amplified products were purified using commercial kits (Fermentas, Lithuania) and used for an additional PCR reaction with primers containing marker sequences designed for the 2x250bp read lengths sequencing protocol, according to the manufacturer’s instructions (Illumina, USA). The following re-amplification profile was used: 3 min at 95 °C; 4 cycles of 15 s at 95 °C, 15 s at 56 °C, and 60 s at 72 °C; 15 cycles of 10 s at 95 °C, 10 s at 55 °C, and 30 s at 72 °C; and an additional elongation phase of 5 min at 72 °C. The obtained PCR fragments for the different samples were mixed and purified by electrophoresis in 1.5% agarose gel. Libraries were constructed in October 2012.

#### NGS sequencing

NGS sequencing of the variable V3-V4 regions of the 16S rRNA gene was performed on MiSeq (Illumina) using the MiSeq reagent kit v.2 (Illumina). Library preparation was done with Nextera DNA sample prep (Illumina). Sequencing was performed at the Faculty of Bioengineering and Bioinformatics, Lomonosov Moscow State University in April 2013.

#### Metagenomic data processing

16S rRNA reads (a total of 232,310 reads) were filtered, denoised, and processed with a QIIME pipeline v1.9.1 [71]. All sequences were clustered, de novo chimera checked and quality filtered using USEARCH (Ultra-fast sequence analysis) tool v5.2.236 [72] against the Gold database (http://drive5.com/uchime/gold.fa). Sequences tagged as non-chimeric were combined and sorted by abundance. Then, operational taxonomic units (OTUs) picking was performed; each non-chimeric read was assigned to a specific OTU identifier. A representative sequence for each OTU was queried against the GREENGENES database v13_8 [73] using UCLUST v1.2.22q program (http://www.drive5.com/uclust) from QIIME.

### Statistical analysis

Statistical analysis was performed using the QIIME – UniFrac software; α- and β-biodiversity, and the Shannon index were calculated.

Statistical comparisons for soil chemical analyses were performed in JMP (SAS Institute, Cary, NC, USA). Repeated-measures analysis of variance (ANOVA) was used on time-series C mineralization measurements, with site (control vs burned), depth and atmosphere (aerobic vs anaerobic) as independent factors and CO_2_ flux as the dependent factor. Two-way ANOVA (site depth) was used to test for differences in soil pH, soil EC and soil C and N. All other statistical tests were produced by using R packages in the R statistical environment (http: //www.r-project.org). Results were significant at *P* < 0.05. To test differences in enzyme activity in the control vs burned samples, one-way ANOVA followed by the Tukey’s honestly significant difference test was used. Ordination of the whole community detected by 16S rRNA gene amplicon sequencing was created from UniFrac matrix calculated by QIIME software and presented in a principal coordinates analysis plot. The contribution of sampling strategy (fire depth) and soil geochemistry to the observed variation in b-diversity was tested via variation partitioning and subsequent ANOVA analysis. Furthermore, principal component analysis (PCA) was performed on Hellinger transformed relative OTU abundances (downweights highly abundant OTUs while avoiding overweighting of rare OTUs). To determine which environmental parameter (i.e. depth, burn, soil geochemistry and total enzyme activity) could significantly explain the variation in bacterial community structure, a canonical redundancy analysis was used, and its significance was assessed by ANOSIM (analysis of similarities; 999 Monte Carlo permutation tests). In cases where the redundancy analysis achieved statistical significance, Pearson’s correlation tests were used to detect significant positive or negative correlations of each OTU abundance distribution with the corresponding environmental variable. Pairwise similarities calculated by Compareads between the metagenomes were visualized by heatmaps and hierarchical clustering. Normalized KO (KEGG ORTHOLOGY) relative abundances were used to explore the correlations between different depths of fire-impacted and control samples. Between-class analysis was used as implemented in ade4 package, where the analysis finds the principal components based on the center of gravity of each group. Significance of this analysis was tested with a subsequent ANOVA. Differences in the relative abundances of C- and N-cycle genes were represented as a heatmap, where data matrix was centered by subtracting the column means and scaled by dividing the (centered) columns by their standard deviation.

Data on bacterial and archaeal proteins (uniprot_trembl_bacteria.dat, uniprot_sprot_bacteria.dat, uniprot_trembl_archaea.dat, uniprot_sprot_archaea.dat) was taken from UniProt (ftp://ftp.uniprot.org/pub/databases/uniprot/current_release/knowledgebase/taxonomic_divisions/). According to the taxonomic annotation, all proteins from the UniProt database for the given species were extracted. For each sampling point, a non-redundant protein dataset combining all species was constructed. The completeness of metabolic pathways was estimated as the ration of the number of detected enzymes involved in a given pathway to the total number of enzymes for this pathway. Enzyme overlap was estimated by KEGG ORTHOLOGY (KO) identifiers. 146 KEGG pathways found in microorganisms were selected manually.

The Principal Component Analysis and 2B-PLS [74] were performed using Statistica 8, Excel, and PAST [75].

## Supplementary information

**Additional file 1: Figure S1.** Eh-pH values of the oil site waters. Lines I, II, III, and IV represent the detected trends. 1, natural outlets; 2, sampling pits from the southwestern part of the field with clay minerals and no visible oil; 3, sampling pits with oil film; 4, sampling pits with large oil drops. Sites with red borders represent the samples for which microbial composition was studied.

**Additional file 2: Table S1.** Water samples. Note: b.d.l. – below detected limit, n.d. – no data.

**Additional file 3: Figure S2.** Ion content of the oil site waters.

**Additional file 4: Table S2.** Percentage of reads in the studied samples (values below 0.05% not shown) based on the number of reads longer than 250 bp.

**Additional file 5: Table S3.** The number of OTUs in the studied communities

**Additional file 6. Table S4.** Percentage of OTUs in the studied samples (values below 0.05% not shown)

**Additional file 7: Figure S3.** Color code for Fig. 1 (main text).

**Additional file 8: Figure S4-A, Additional file 7: Fig. S4-B.** Completeness of the naphthalene degradation (А) and benzoate degradation (B) metabolic pathways for the U3.4.10 sample. Red borders indicate the proteins found in U3.4.10 sample. Visualization was made with ANDVisio program of ANDSystem (www.bionet.sscc.ru/and/cell/#!/app/andvisio).

**Additional file 9: Figure S5.** Completeness of the Ethylbenzene degradation metabolic pathway for the U3.4.10 and U3kot samples. Red borders indicate the proteins found in U3kot; purple, in U3.4.10. Visualization was made with ANDVisio program of ANDSystem (www.bionet.sscc.ru/and/cell/#!/app/andvisio).

**Additional file 10: Figure S6.** The Uzon oil site. Left, Yashcheritsa spring (natural outlet, 3 m diameter); right, U3_2–3 sampling pit (0.3 m diameter).

**Additional file 11: Table S5.** Detection limits of elements for ICP-MS, ppb.

## Data Availability

All data generated or analysed during this study are included in this published article and its supplementary information files. All datasets analyzed in the study are available in the Sequence Read Archive (SRA) repository, under the following accession numbers: SRR11496424, SRR11496423, SRR11496431, SRR11496429, SRR11496425, SRR11496428, SRR11496432, SRR11496430, SRR11496426, SRR11496427.

## Abbreviations

OTUs: Operational taxonomic units
KEGG: Kyoto encyclopedia of genes and genomes
PTS: Phosphotransferase system
2B-PLS: Two-block partial least-squares
TDS: Total dissolved solids
NGS: Next generation sequencing
ICP-MS: Mass spectrometry with inductively coupled plasma
EDTA: Ethylenediaminetetraacetic acid
PCR: Polymerase chain reaction
DMSO: Dimethyl sulfoxide
EC: Electrical conductivity
PCA: Principal component analysis
PAST: Paleontological statistics

## References

[1] Simoneit BRT, Kvenvolden KA (1994). Comparison of14C ages of hydrothermal petroleums. Org Geochem.

[2] Dobretsov NL, Lazareva EV, Zhmodik SM, Bryanskaya AV, Morozova VV, Tikunova NV (2015). Geological, hydrogeochemical, and microbiological characteristics of the oil site of the Uzon caldera (Kamchatka). Russ Geol Geophys.

[3] Didyk BM, Simoneit BRT (1989). Hydrothermal oil of Guaymas Basin and implications for petroleum formation mechanisms. Nature..

[4] Clifton CG, Walters CC, Simoneit BRT (1990). Hydrothermal petroleums from Yellowstone National Park, Wyoming, USA. Appl Geochem.

[5] Simoneit BRT, Aboul-Kassim TAT, Tiercelin JJ (2000). Hydrothermal petroleum from lacustrine sedimentary organic matter in the east African rift. Appl Geochem.

[6] Simoneit BRT, Deamer DW, Kompanichenko V (2009). Characterization of hydrothermally generated oil from the Uzon caldera, Kamchatka. Appl Geochem.

[7] Varfolomeev SD, Karpov GA, Synal H-A, Lomakin SM, Nikolaev EN (2011). The youngest natural oil on earth. Dokl Chem.

[8] Beskrovnyi NS, Lebedev BA (1971). Oil seep in the Uzon caldera of Kamchatka. Dokl Akad Nauk.

[9] Kalinko MK (1975). Genesis of oil microshows of the Uzon caldera (eastern Kamchatka). Transformation of organic matter in the recent and fossil sediments and the major stages of generation of free hydrocarbons.

[10] Bazhenova OK, Arefiev OA, Frolov EB (1998). Oil of the volcano Uzon caldera, Kamchatka. Org Geochem.

[11] Kontorovich AE, Bortnikova SB, Karpov GA, Kashirtsev VA, Kostyreva EA, Fomin AN (2011). Uzon volcano caldera (Kamchatka): a unique natural laboratory of the present-day naphthide genesis. Russ Geol Geophys.

[12] Fursenko EA, Kashirtsev VA, Kontorovich AE, Fomin AN (2014). Naphthides of continental hydrotherms (Uzon, Yellowstone, New Zealand): geochemistry and genesis. Russ Geol Geophys.

[13] Holm NG, Cairns-Smith AG, Daniel RM, Ferris JP, Hennet RJ, Shock E (1992). Marine hydrothermal systems and the origin of life. Orig Life Evol Biosph.

[14] Holm NG, Charlou JL (2001). Initial indications of abiotic formation of hydrocarbons in the rainbow ultramafic hydrothermal system, mid-Atlantic ridge. Earth Planet Sci Lett.

[15] Foustoukos DI, Seyfried WE (2004). Hydrocarbons in hydrothermal vent fluids: the role of chromium-bearing catalysts. Science..

[16] Simoneit BRT (1990). Petroleum generation, an easy and widespread process in hydrothermal systems: an overview. Appl Geochem.

[17] Weston RJ, Woolhouse AD (1987). Organic geochemistry of the sedimentary basins of New Zealand part IV. A biomarker study of the petroleum seepage and some well core bitumens from the geothermal region of Ngawha Springs. Appl Geochem.

[18] Czochanska Z, Sheppard CM, Weston RJ, Woolhouse AD, Cook RA (1986). Organic geochemistry of sediments in New Zealand. Part I. a biomarker study of the petroleum seepage at the geothermal region of Waiotapu. Geochim Cosmochim Acta.

[19] Yamanaka T, Ishibashi J, Hashimoto J (2000). Organic geochemistry of hydrothermal petroleum generated in the submarine Wakamiko caldera, southern Kyushu, Japan. Org Geochem.

[20] Leonov VL, Grib EN (1998). Calderas and ignimbrites of Uzon-Semiachik area, Kamchatka. Vulkanol i Seismol.

[21] Fedotov SA (1991). Active volcanoes of Kamchatka.

[22] Bychkov AY (2009). A geochemical model of contemporary ore formation in the Uzon caldera (Kamchatka).

[23] Egorova IA (1993). Age and paleogeography of formation of volcano-sedimentary deposits in the Uzon-Geizernaya caldera depression, Kamchatka (according to Palyological data). Volcanol Seismol.

[24] Subsoil M (1974). Volcanism, hydrothermal process and ore formation.

[25] Migdisov AA, Bychkov AY (1998). The behaviour of metals and Sulphur during the formation of hydrothermal mercury-antimony-arsenic mineralization, Uzon caldera, Kamchatka, Russia. J Volcanol Geotherm Res.

[26] Karpov GS (1988). Modern Hydrotherms and hg–Sb–as mineralization.

[27] Beskrovnyi NS, Glavatskikh SF, Lebedev BA, Naboko SI, Chegletsova EA (1970). Metals and oil in hydrothermal solutions of the Uzon caldera. Modern metal-forming solutions.

[28] Lukin AE, Pikovskij YI (2004). New data on isotope composition of the hydrothermal oil (the caldera Uzon at Kamchatka). Dokl Akad Nauk.

[29] Karpov G, Bonch-Osmolovskaya E, Zavarzin G, Lupikina EG (2008). To the characteristic of thermophilic microorganisms of the Uzon caldera (eastern Kamchatka): conservation of the biodiversity of Kamchatka and adjacent seas.

[30] Gradova NB, Gornova IB, Eddaudi R, Salina RN (2003). Use of bacteria of the genus Azotobacter for bioremediation of oil-contaminated soils. Appl Biochem Microbiol.

[31] Rahman KSM, Rahman T, Lakshmanaperumalsamy P, Banat IM (2002). Occurrence of crude oil degrading bacteria in gasoline and diesel station soils. J Basic Microbiol.

[32] Stabnikova EV, Selezneva MV, Ivanov VN, Reva O (1995). Theoretical and experimental screening of microbial component of biopreparation using for bioremediation of soil contaminated with oil. Appl Biochem Microbiol.

[33] Gumerov VM, Mardanov AV, Beletsky AV, Bonch-Osmolovskaya EA, Ravin NV (2011). Molecular analysis of microbial diversity in the Zavarzin spring, Uzon caldera, Kamchatka. Microbiology.

[34] Lobkova LE, Lobkov EG (2003). The role of biological components in the ecosystems of the Uzon and Geyser Valley thermal fields and some aspects of the protection of thermal biogeocenoses: preserving the biodiversity of Kamchatka and adjacent seas.

[35] Mardanov AV, Gumerov VM, Beletsky AV, Perevalova AA, Karpov GA, Bonch-Osmolovskaya EA (2011). Uncultured archaea dominate in the thermal groundwater of Uzon caldera, Kamchatka. Extremophiles.

[36] Kochetkova TV, Rusanov II, Pimenov NV, Kolganova TV, Lebedinsky AV, Bonch-Osmolovskaya EA (2011). Anaerobic transformation of carbon monoxide by microbial communities of Kamchatka hot springs. Extremophiles..

[37] Perevalova AA, Svetlichny VA, Kublanov IV, Chernyh NA, Kostrikina NA, Tourova TP (2005). *Desulfurococcus fermentans* sp. nov., a novel hyperthermophilic archaeon from a Kamchatka hot spring, and emended description of the genus *Desulfurococcus*. Int J Syst Evol Microbiol.

[38] Slepova TV, Sokolova TG, Lysenko AM, Tourova TP, Kolganova TV, Kamzolkina OV (2006). *Carboxydocella sporoproducens* sp. nov., a novel anaerobic CO-utilizing/H2-producing thermophilic bacterium from a Kamchatka hot spring. Int J Syst Evol Microbiol.

[39] Gumerov VM (2011). Molecular analysis of microbial biodiversity of hot springs of Kamchatka.

[40] Chernyh NA, Mardanov AV, Gumerov VM, Miroshnichenko ML, Lebedinsky AV, Merkel AY (2015). Microbial life in Bourlyashchy, the hottest thermal pool of Uzon caldera, Kamchatka. Extremophiles..

[41] Slutskaya ES, Bezsudnova EY, Mardanov AV, Gumerov VM, Rakitina TV, Popov VO (2012). Characteristics of the new M42 aminopeptidase from the crenarchaea of *Desulfurococcus kamchatkensis*. Dokl Akad Nauk.

[42] Zenova GM, Manucharova NA, Zvyagintsev DG (2011). Extremophilic and extremotolerant actinomycetes in different soil types. Eurasian Soil Sci.

[43] Petushkova YP, Lyalikova NN, Poglazova MN (1989). Microorganisms found on the Ferapont (Vologda oblast, Russian S.F.S.R., U.S.S.R.) monastery frescoes. Microbiology.

[44] Berdichevskaya MV, Kozyreva GI, Blaginykh AV (1991). The size, species composition, and oxygenase activity of the hydrocarbon-oxidizing community of oil-polluted Ural and westem Siberia rivers. Microbiology..

[45] Greer CW, van Beilen JB, Labbe D, Smits THM, Whyte LG, Witholt B (2002). Gene cloning and characterization of multiple alkane hydroxylase systems in Rhodococcus strains Q15 and NRRL B-16531. Appl Environ Microbiol.

[46] Amouric A, Quéméneur M, Grossi V, Liebgott PP, Auria R, Casalot L (2010). Identification of different alkane hydroxylase systems in Rhodococcus ruber strain SP2B, an hexane-degrading actinomycete. J Appl Microbiol.

[47] Sorkhoh NA, Ghannoum MA, Ibrahim AS, Stretton RJ, Radwan SS (1990). Crude oil and hydrocarbon-degrading strains of Rhodococcus rhodochrous isolated from soil and marine environments in Kuwait. Environ Pollut.

[48] Lechevalier MP, Lechevalier H (1985). Biology of actinomycetes not belonging to the genus Streptomyces. Biotechnol Ser.

[49] Nakajima K, Sato A, Takahara Y, Iida T (1985). Microbial oxidation of isoprenoid alkanes, phytane, norpristane and farnesanet. Agric Biol Chem.

[50] Martínková L, Uhnáková B, Pátek M, Nešvera J, Křen V (2009). Biodegradation potential of the genus Rhodococcus. Environ Int.

[51] Baptist JN, Gholson RK, Coon MJ (1963). Hydrocarbon oxidation by a bacterial enzyme system. I. Products of octane oxidation. Biochim Biophys Acta.

[52] van Beilen JB, Kingma J, Witholt B (1994). Substrate specificity of the alkane hydroxylase system of pseudomonas oleovorans GPo1. Enzyme Microb Technol.

[53] Grant C, Woodley JM, Baganz F (2011). Whole-cell bio-oxidation of n-dodecane using the alkane hydroxylase system of P. putida GPo1 expressed in *E. coli*. Enzyme Microb Technol.

[54] Kersters K, Devos P, Gillis M, Swings J, Vandamme P, Stackebrandt E (2006). The prokaryotes: a handbook on the biology of bacteria.

[55] Joseph TC, Baby A, Reghunathan D, Varghese AM, Murugadas V, Lalitha KV (2014). Draft genome sequence of the Halophilic and highly Halotolerant Gammaproteobacteria strain MFB021. Genome Announc.

[56] Kato T, Haruki M, Imanaka T, Morikawa M, Kanaya S (2001). Isolation and characterization of long-chain-alkane degrading bacillus thermoleovorans from deep subterranean petroleum reservoirs. J Biosci Bioeng.

[57] Tourova TP, Nazina TN, Mikhailova EM, Rodionova TA, Ekimov AN, Mashukova AV (2008). alkB homologs in thermophilic bacteria of the genus Geobacillus. Mol Biol.

[58] Golovleva LA (2014). Bioremediation of soils contaminated by pollutants. Ann Agrarian Sci.

[59] Baraniecki CA, Aislabie J, Foght JM (2002). Characterization of *Sphingomonas* sp. ant 17, an aromatic hydrocarbon-degrading bacterium isolated from Antarctic soil. Microb Ecol.

[60] Johnsen AR, Wick LY, Harms H (2005). Principles of microbial PAH-degradation in soil. Environ Pollut.

[61] Bacosa HP, Suto K, Inoue C (2013). Degradation potential and microbial community structure of heavy oil-enriched microbial consortia from mangrove sediments in Okinawa, Japan. J Environ Sci Health.

[62] Weekers F, Thonart P, Jacques P, Springael D, Mergeay M, Diels L (1998). Effect of drying on bioremediation bacteria PropertiesBiotechnology for fuels and chemicals.

[63] Timergazina IF, Perekhodova LS (2012). To the problem of the biological oxidation of oil and oil products by hydrocarbon oxidizing microorganisms. Petroleum Geol Theory Pract.

[64] Takai K, Nakagawa S (2014). The family Hydrogenothermaceae.

[65] Shestakov SV (2012). Impact of metagenomics on biotechnological development. Appl Biochem Microbiol.

[66] Wagner ID, Zhao W, Zhang CL, Romanek CS, Rohde M, Wiegel J (2008). Thermoanaerobacter uzonensis sp. nov., an anaerobic thermophilic bacterium isolated from a hot spring within the Uzon caldera, Kamchatka, Far East Russia. Int J Syst Evol Microbiol.

[67] Wagner ID, Varghese LB, Hemme CL, Wiegel J (2013). Multilocus sequence analysis of thermoanaerobacter isolates reveals recombining, but differentiated, populations from geothermal springs of the Uzon caldera, Kamchatka, Russia. Front Microbiol.

[68] Inskeep WP, Rusch DB, Jay ZJ, Herrgard MJ, Kozubal MA, Richardson TH (2010). Metagenomes from high-temperature chemotrophic systems reveal geochemical controls on microbial community structure and function. PLoS One.

[69] Inskeep WP, Jay ZJ, Tringe SG, Herrgård MJ, Rusch DB (2013). The YNP metagenome project: environmental parameters responsible for microbial distribution in the Yellowstone geothermal ecosystem. Front Microbiol.

[70] Burgess EA, Unrine JM, Mills GL, Romanek CS, Wiegel J (2012). Comparative geochemical and microbiological characterization of two thermal pools in the Uzon caldera, Kamchatka, Russia. Microb Ecol.

[71] Caporaso JG, Kuczynski J, Stombaugh J, Bittinger K, Bushman FD, Costello EK (2010). QIIME allows analysis of high-throughput community sequencing data. Nat Methods.

[72] Edgar RC (2010). Search and clustering orders of magnitude faster than BLAST. Bioinformatics..

[73] DeSantis TZ, Hugenholtz P, Larsen N, Rojas M, Brodie EL, Keller K (2006). Greengenes, a chimera-checked 16S rRNA gene database and workbench compatible with ARB. Appl Environ Microbiol.

[74] Abdi H (2010). Partial least squares regression and projection on latent structure regression (PLS regression). Wiley Interdiscip Rev Comput Stat.

[75] Hammer Ø, Harper DAT, Ryan PD (2001). Past: paleontological statistics software package for education and data analysis. Palaeontol Electron.

