# Supplementary figures and images for: Young «oil site» of the Uzon Caldera as a habitat for unique microbial life
Source: BMC Microbiol. 2020 Nov 24;20(Suppl 2):349. doi: 10.1186/s12866-020-02012-1 (PMC7685581; doi:10.1186/s12866-020-02012-1)

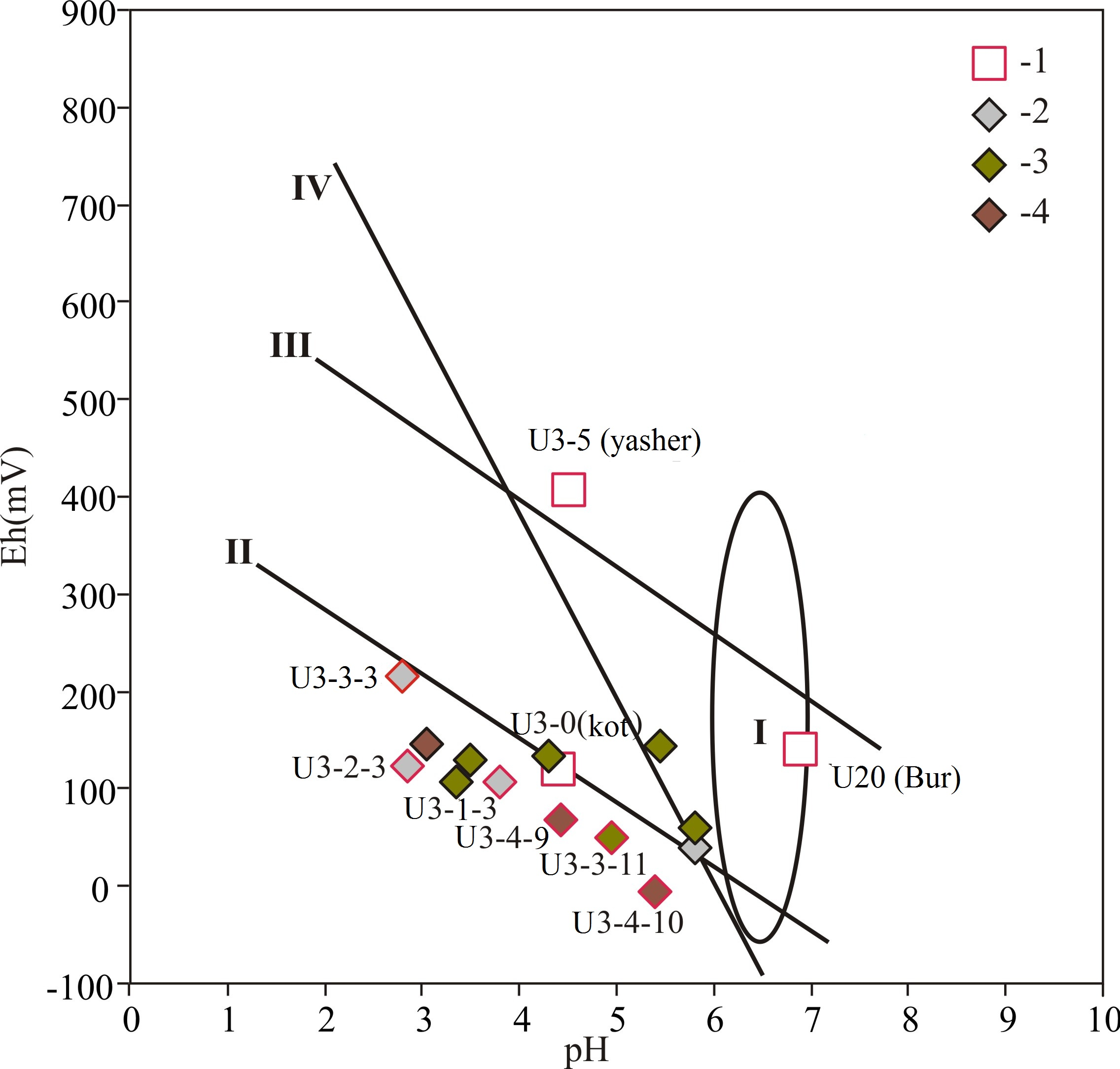

Supplement: Supplementary file 1 — Additional file 1: Figure S1. Eh-pH values of the oil site waters. Lines I, II, III, and IV represent the detected trends. 1, natural outlets; 2, sampling pits from the southwestern part of the field with clay minerals and no visible oil; 3, sampling pits with oil film; 4, sampling pits with large oil drops. Sites with red borders represent the samples for which microbial composition was studied. [file 12866_2020_2012_MOESM1_ESM.png]

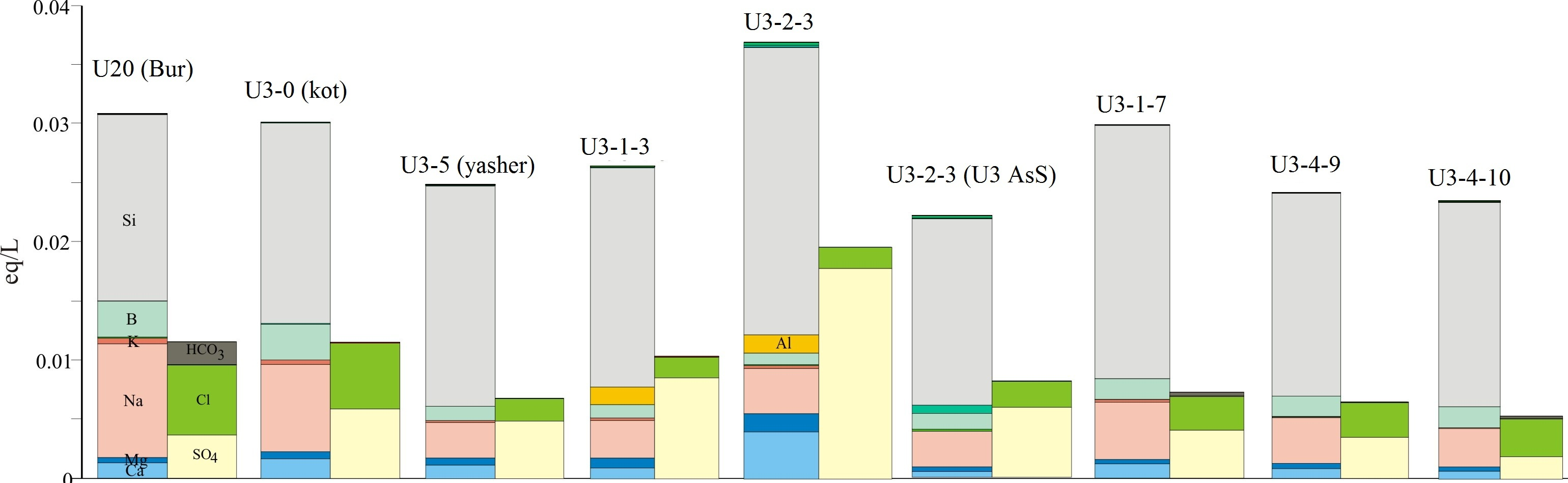

Supplement: Supplementary file 3 — Additional file 3: Figure S2. Ion content of the oil site waters. [file 12866_2020_2012_MOESM3_ESM.png]

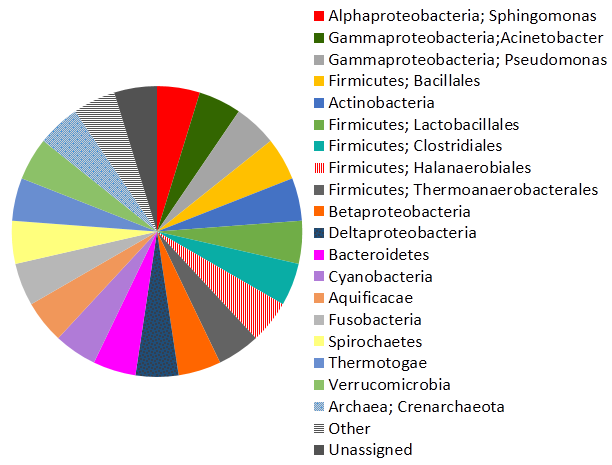

Supplement: Supplementary file 7 — Additional file 7: Figure S3. Color code for Fig. 1 (main text). [file 12866_2020_2012_MOESM7_ESM.png]

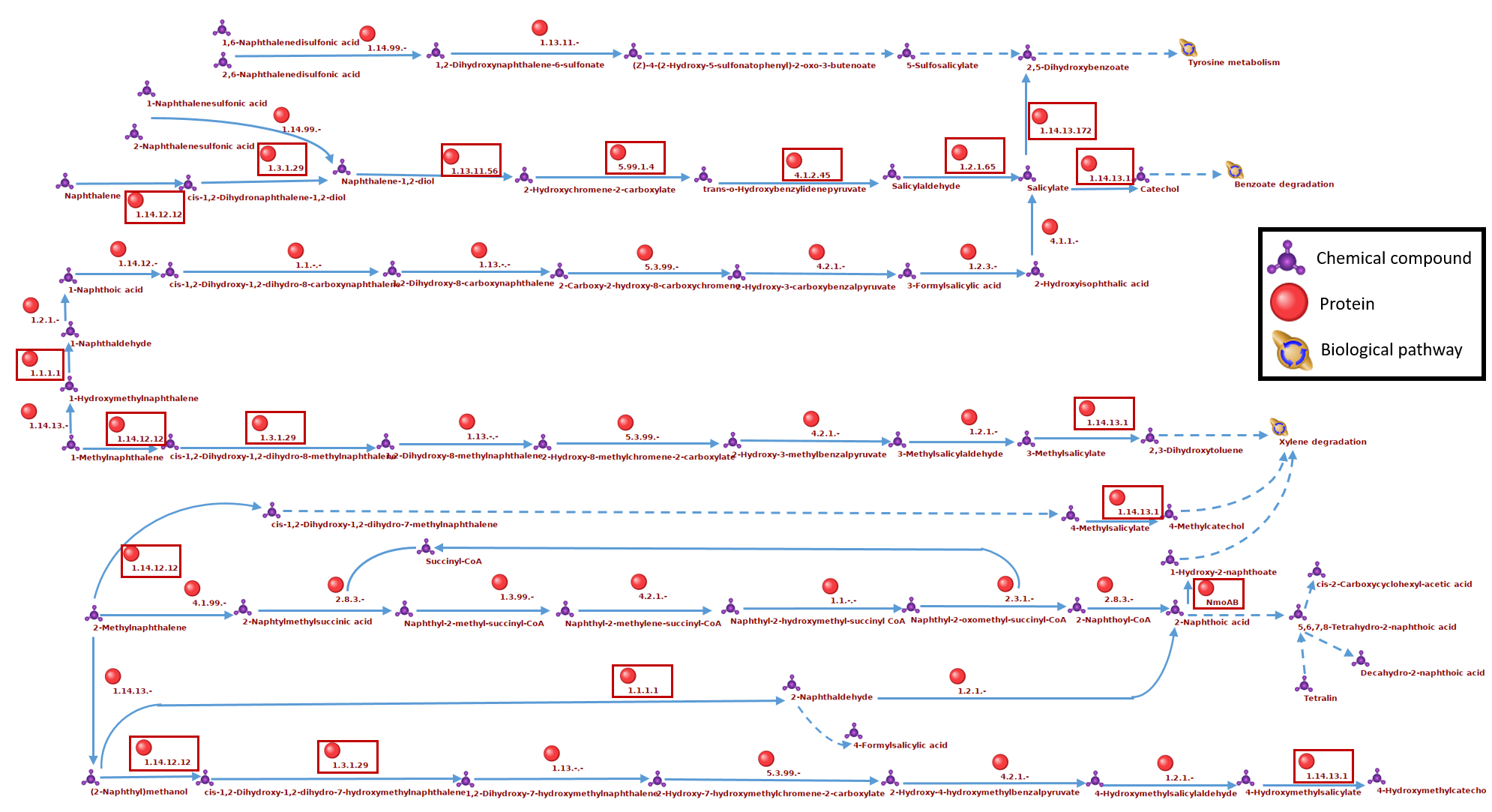

Supplement: Supplementary file 8 — Additional file 8: Figure S4-A, Additional file 7: Fig. S4-B. Completeness of the naphthalene degradation (А) and benzoate degradation (B) metabolic pathways for the U3.4.10 sample. Red borders indicate the proteins found in U3.4.10 sample. Visualization was made with ANDVisio program of ANDSystem (www.bionet.sscc.ru/and/cell/#!/app/andvisio). [file 12866_2020_2012_MOESM8_ESM.zip › Addit_File8_Fig_S4-A.png]

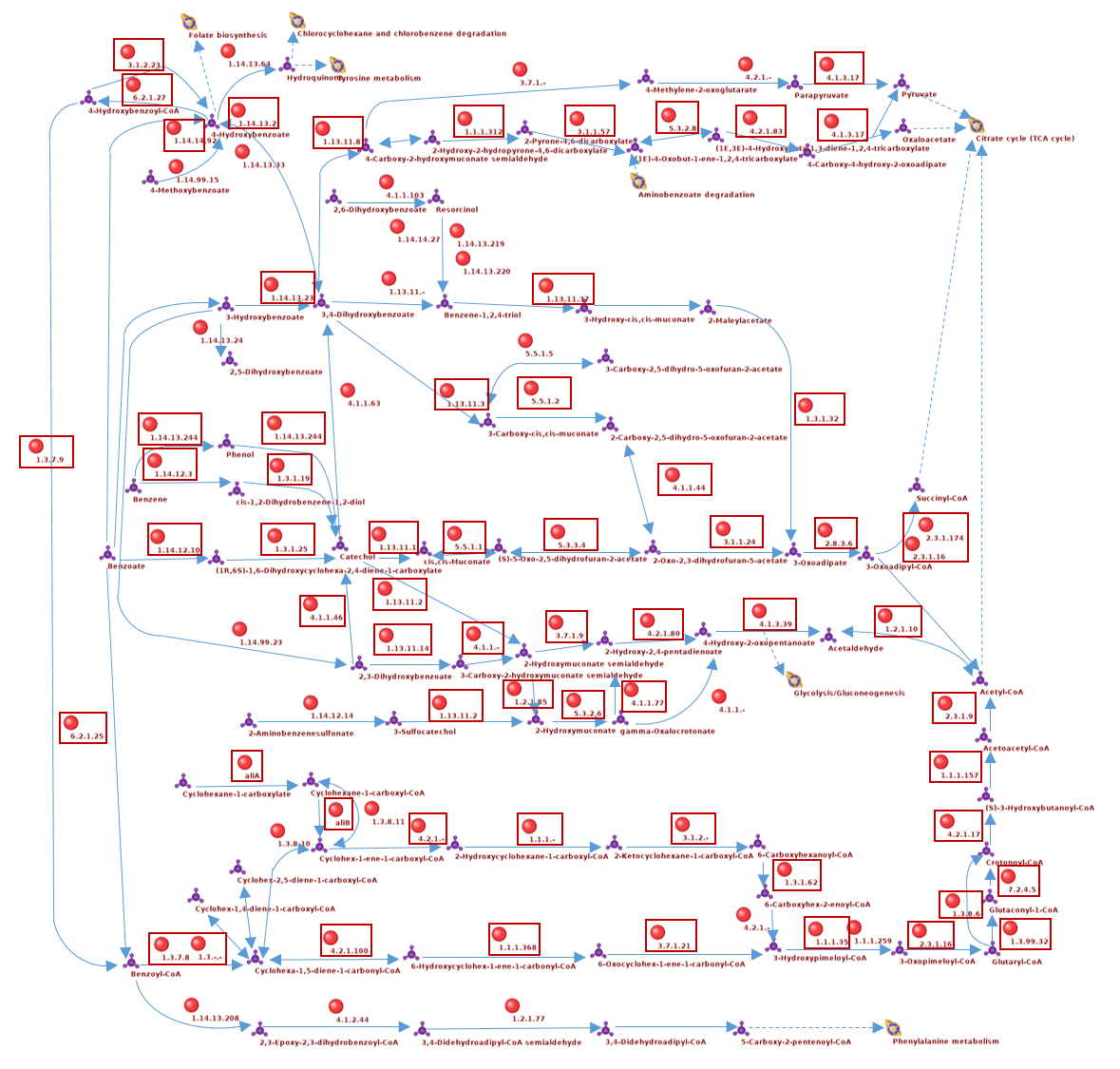

Supplement: Supplementary file 8 — Additional file 8: Figure S4-A, Additional file 7: Fig. S4-B. Completeness of the naphthalene degradation (А) and benzoate degradation (B) metabolic pathways for the U3.4.10 sample. Red borders indicate the proteins found in U3.4.10 sample. Visualization was made with ANDVisio program of ANDSystem (www.bionet.sscc.ru/and/cell/#!/app/andvisio). [file 12866_2020_2012_MOESM8_ESM.zip › Addit_File8_Fig_S4-B.png]

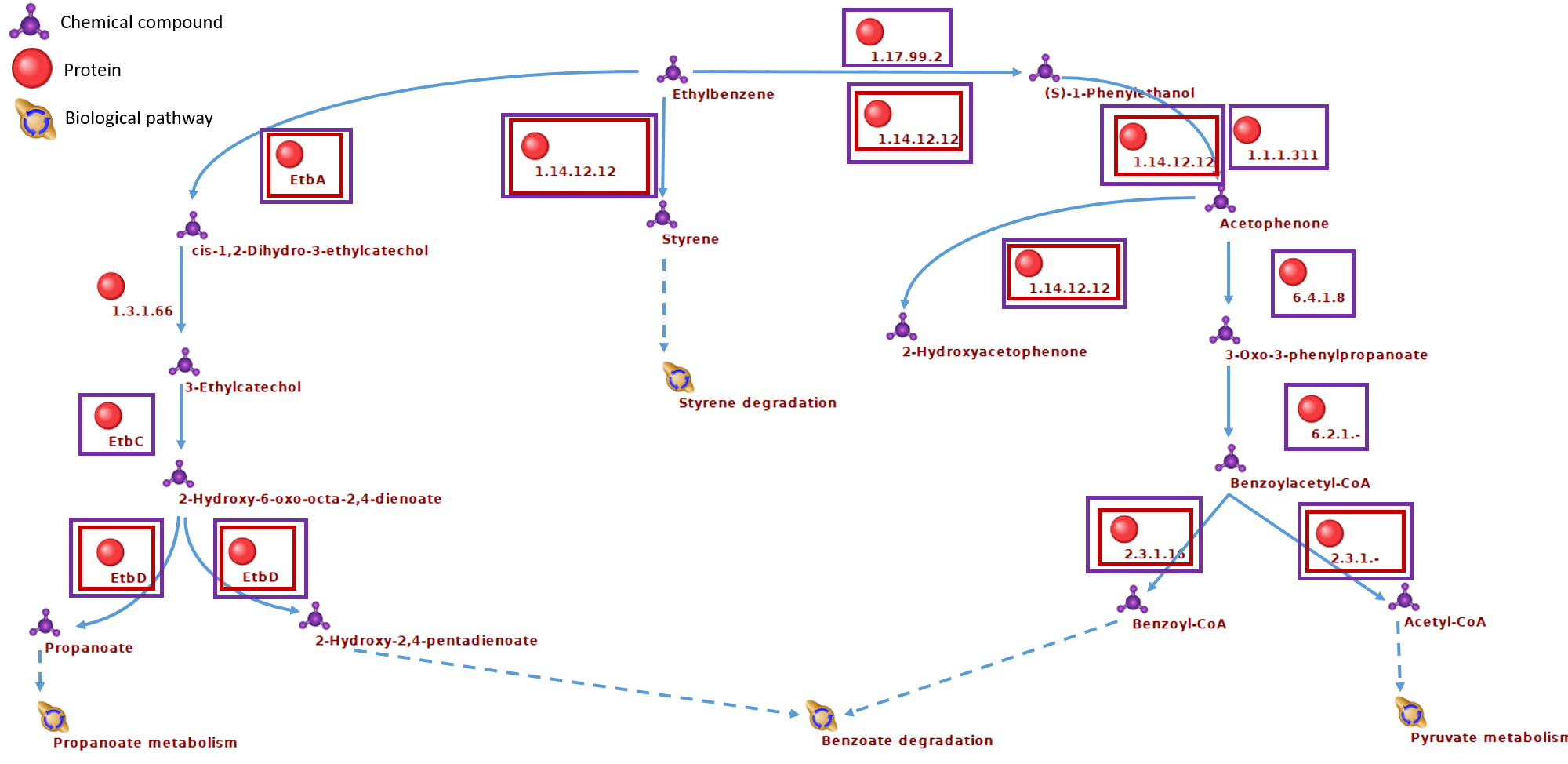

Supplement: Supplementary file 9 — Additional file 9: Figure S5. Completeness of the Ethylbenzene degradation metabolic pathway for the U3.4.10 and U3kot samples. Red borders indicate the proteins found in U3kot; purple, in U3.4.10. Visualization was made with ANDVisio program of ANDSystem (www.bionet.sscc.ru/and/cell/#!/app/andvisio). [file 12866_2020_2012_MOESM9_ESM.png]

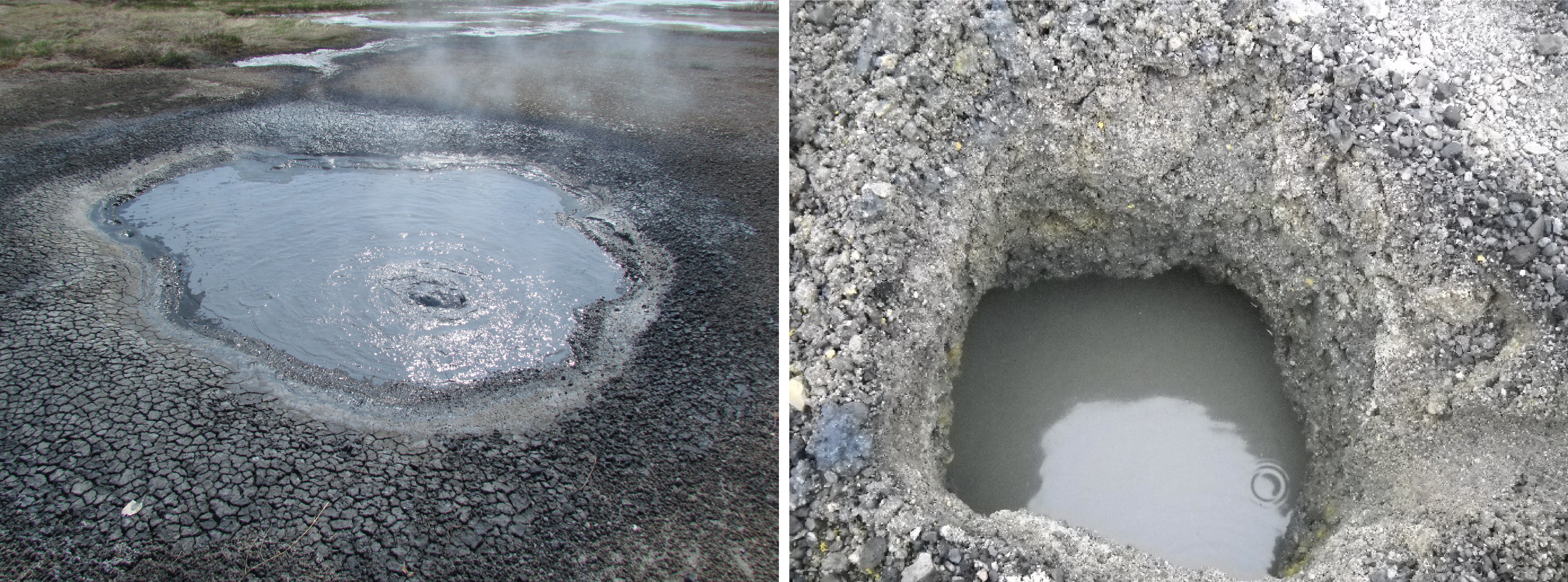

Supplement: Supplementary file 10 — Additional file 10: Figure S6. The Uzon oil site. Left, Yashcheritsa spring (natural outlet, 3 m diameter); right, U3_2–3 sampling pit (0.3 m diameter). [file 12866_2020_2012_MOESM10_ESM.png]
