# Supplementary material for: Young «oil site» of the Uzon Caldera as a habitat for unique microbial life
Source: BMC Microbiol. 2020 Nov 24;20(Suppl 2):349. doi: 10.1186/s12866-020-02012-1 (PMC7685581; doi:10.1186/s12866-020-02012-1)
Supplement: Supplementary file 4 — Additional file 4: Table S2. Percentage of reads in the studied samples (values below 0.05% not shown) based on the number of reads longer than 250 bp. [file 12866_2020_2012_MOESM4_ESM.docx]

Table S2. Percentage of reads in the studied samples (values below 0.05% not shown) based on the number of reads longer than 250 bp.

| Taxon | U3_1-3 | U3_2-3 | U3_  4-9 | U3_  4-10 | U3AS | U3  kot | U3_5 yasher | U20 Bur | U5_1 | G11-1а |
| --- | --- | --- | --- | --- | --- | --- | --- | --- | --- | --- |
| Proteobacteriа/  Alphaproteobacteriа/  Sphingomonas | 5.7 | 11.1 | 9.2 | 17.9 | 3.8 | 2.2 | 12.0 | 5.70 | 0.46 |  |
| Proteobacteriа/ Betaproteobacteria | 1.1 | 1,6 | 3.5 | 3.6 | 1.7 | 22.9 | 1.3 | 5.92 | 0.20 | 3.37 |
| Proteobacteriа/  Gammaproteobacteria/  Acinetobacter |  |  |  |  |  |  |  | 0.36 |  | 0.41 |
| Proteobacteriа/  Gammaproteobacteria/  Pseudomonas | 12.6 | 3.4 | 20.9 | 21.5 | 21.7 | 4.7 | 8.6 | 0.59 | 0.34 | 0.12 |
| Proteobacteriа/  Deltaproteobacteria |  | 29.4 | 1.2 | 4.5 |  |  | 5.9 | 7.80 | 14.78 | 0.09 |
| Actinobacteria | 57.8 | 2.3 | 32.5 | 16.3 | 28.2 | 47.0 | 10.6 | 33.74 | 0.06 | 46.72 |
| Firmicutes/Bacillales |  |  | 14.8 | 4.0 | 3.3 |  | 53.3 | 3.49 | 1.21 | 3.49 |
| Firmicutes/  Lactobacillales | 8.8 |  | 2.3 | 4.1 | 21.8 | 18.1 |  | 0.51 |  | 0.11 |
| Firmicutes/Clostridiales |  |  |  | 1.9 | 5.2 |  |  | 0.62 |  | 1.82 |
| Firmicutes/  Halanaerobiales |  | 2.8 |  |  |  |  |  |  | 0.95 |  |
| Firmicutes; Thermoanaerobacterales |  |  |  | 2.5 |  |  |  |  |  |  |
| Bacteroidetes | 3.5 | 20.0 |  | 7.8 |  | 1.9 |  | 1.32 | 3.84 | 1.47 |
| Cyanobacteria |  | 12.9 | 3.1 | 3.6 | 6.7 |  | 1.2 | 7.90 | 3.32 | 9.06 |
| Aquificae |  |  |  |  |  |  | 1.5 |  |  |  |
| Fusobacteria |  |  |  | 1.9 |  |  |  |  |  |  |
| Spirochaetes |  | 3.2 |  |  |  |  |  |  | 0.89 | 0.08 |
| Thermotogae |  |  |  |  |  |  |  |  | 0.05 |  |
| Verrucomicrobia |  |  | 2.9 |  |  |  |  | 5.00 | 0.13 | 3.67 |
| Archaea; Crenarchaeota |  |  |  |  |  |  | 1.2 | 0.57 | 1.07 |  |
| Other | 9.4 | 7.3 | 7.1 | 6.3 | 5.1 | 1.8 | 3.3 | 13.63 | 56.14 | 17.79 |
| Unassigned | 1.0 | 5.9 | 2.4 | 4.2 | 2.6 | 1.5 | 1.2 | 12.85 | 16.66 | 11.39 |
