# Supplementary material for: Young «oil site» of the Uzon Caldera as a habitat for unique microbial life
Source: BMC Microbiol. 2020 Nov 24;20(Suppl 2):349. doi: 10.1186/s12866-020-02012-1 (PMC7685581; doi:10.1186/s12866-020-02012-1)
Supplement: Supplementary file 5 — Additional file 5: Table S3. The number of OTUs in the studied communities [file 12866_2020_2012_MOESM5_ESM.docx]

Table S3. The number of OTUs in the studied communities

| Taxon | U3_  1-3 | U3_  2-3 | U3_  4-9 | U3_  4-10 | U3AS | U3kot | U3_5  yasher | UBur | U5-1 | G11-1а |
| --- | --- | --- | --- | --- | --- | --- | --- | --- | --- | --- |
| Alphaproteobacteriа; Sphingomonas | 2 | 1 | 1 | 3 | 1 | 1 | 2 | 2 | 1 |  |
| Betaproteobacteria | 6 | 4 | 11 | 8 | 3 | 6 | 8 | 8 | 1 | 7 |
| Gammaproteobacteria; Acinetobacter |  |  |  |  |  |  |  | 2 |  | 2 |
| Gammaproteobacteria; Pseudomonas | 6 | 2 | 3 | 2 | 7 | 3 | 4 | 2 | 1 | 1 |
| Deltaproteobacteria |  | 20 | 6 | 7 |  |  | 3 | 6 | 16 | 1 |
| Actinobacteria | 15 | 8 | 25 | 18 | 22 | 11 | 19 | 22 | 1 | 28 |
| Firmicutes; Bacillales |  |  | 8 | 10 | 3 |  | 9 | 6 | 1 | 7 |
| Firmicutes; Lactobacillales | 5 |  | 6 | 5 | 6 | 4 |  | 2 |  | 1 |
| Firmicutes; Clostridiales |  |  |  | 11 | 9 |  |  | 5 |  | 4 |
| Firmicutes; Halanaerobiales |  | 5 |  |  |  |  |  |  | 3 |  |
| Firmicutes; Thermoanaerobacterales |  |  |  | 4 |  |  |  |  |  |  |
| Bacteroidetes | 7 | 32 |  | 7 |  | 2 |  | 11 | 16 | 11 |
| Cyanobacteria |  | 6 | 16 | 5 | 7 |  | 11 | 14 | 3 | 13 |
| Aquificae |  |  |  |  |  |  | 3 |  |  |  |
| Fusobacteria |  |  |  | 2 |  |  |  |  |  |  |
| Spirochaetes |  | 17 |  |  |  |  |  |  | 5 | 1 |
| Thermotogae |  |  |  |  |  |  |  |  | 1 |  |
| Verrucomicrobia |  |  | 12 |  |  |  |  | 8 | 2 | 7 |
| Archaea; Crenarchaeota |  |  |  |  |  |  | 12 | 5 | 5 |  |
| Other | 47 | 51 | 87 | 45 | 33 | 20 | 45 | 33 | 34 | 40 |
| Unassigned | 14 | 31 | 31 | 23 | 15 | 8 | 14 | 78 | 16 | 55 |
| Total | 102 | 177 | 206 | 150 | 106 | 55 | 130 | 204 | 106 | 178 |
