# Supplementary material for: Young «oil site» of the Uzon Caldera as a habitat for unique microbial life
Source: BMC Microbiol. 2020 Nov 24;20(Suppl 2):349. doi: 10.1186/s12866-020-02012-1 (PMC7685581; doi:10.1186/s12866-020-02012-1)
Supplement: Supplementary file 6 — Additional file 6. Table S4. Percentage of OTUs in the studied samples (values below 0.05% not shown) [file 12866_2020_2012_MOESM6_ESM.docx]

Table S4. Percentage of OTUs in the studied samples (values below 0.05% not shown)

| Taxon | U3_  1-3 | U3_  2-3 | U3_  4-9 | U3_  4-10 | U3AS | U3kot | U3_5  yasher | U20 Bur | U5_1 | G11-1а |
| --- | --- | --- | --- | --- | --- | --- | --- | --- | --- | --- |
| Alphaproteobacteriа; Sphingomonas | 1.96 | 0.57 | 0.49 | 2 | 0.94 | 1.82 | 1.54 | 5.70 | 0.46 |  |
| Betaproteobacteria | 5.88 | 2.26 | 5.34 | 5.33 | 2.83 | 10.91 | 6.15 | 5.92 | 0.20 | 3.88 |
| Gammaproteobacteria;  Acinetobacter |  |  |  |  |  |  |  | 0.36 |  | 0.41 |
| Gammaproteobacteria; Pseudomonas | 5.88 | 1.13 | 1.46 | 1.33 | 6.60 | 5.46 | 3.08 | 0.59 | 0.34 | 0.13 |
| Deltaproteobacteria |  | 11.30 | 2.91 | 4.67 |  |  | 2.31 | 7.79 | 14.69 | 0.09 |
| Actinobacteria | 14.71 | 4.52 | 12.14 | 12 | 20.76 | 20 | 14.62 | 33.74 | 0.06 | 46.72 |
| Firmicutes; Bacillales |  |  | 3.88 | 6.67 | 2.83 |  | 6.92 | 2.98 | 1.21 | 3.37 |
| Firmicutes; Lactobacillales | 4.90 |  | 2.91 | 3.33 | 5.66 | 7.27 |  | 0.51 |  | 0.12 |
| Firmicutes; Clostridiales |  |  |  | 7.33 | 8.49 |  |  | 0.63 |  | 1.83 |
| Firmicutes; Halanaerobiales |  | 2.83 |  |  |  |  |  |  | 0.95 |  |
| Firmicutes; Thermoanaerobacterales |  |  |  | 2.67 |  |  |  |  |  |  |
| Bacteroidetes | 6.86 | 18.08 |  | 4.67 |  | 3.64 |  | 1.32 | 3.84 | 1.47 |
| Cyanobacteria |  | 3.39 | 7.77 | 3.33 | 6.60 |  | 8.46 | 7.91 | 3.32 | 9.06 |
| Aquificae |  |  |  |  |  |  | 2.31 |  |  |  |
| Fusobacteria |  |  |  | 1.33 |  |  |  |  |  |  |
| Spirochaetes |  | 9.61 |  |  |  |  |  |  | 0.89 | 0.08 |
| Thermotogae |  |  |  |  |  |  |  |  | 0.05 |  |
| Verrucomicrobia |  |  | 5.83 |  |  |  |  | 4.99 | 0.13 | 3.67 |
| Archaea; Crenarchaeota |  |  |  |  |  |  | 9.23 | 0.57 | 1.07 |  |
| Other (miror) | 46.08 | 28.81 | 42.23 | 30 | 31.13 | 36.36 | 34.62 | 14.14 | 56.14 | 17.79 |
| Unassigned | 13.73 | 17.51 | 15.05 | 15.33 | 14.15 | 14.55 | 10.77 | 12.86 | 16.66 | 11.39 |
| Total | 100 | 100 | 100 | 100 | 100 | 100 | 100 | 100 | 100 | 100 |
