# Supplementary material for: Young «oil site» of the Uzon Caldera as a habitat for unique microbial life
Source: BMC Microbiol. 2020 Nov 24;20(Suppl 2):349. doi: 10.1186/s12866-020-02012-1 (PMC7685581; doi:10.1186/s12866-020-02012-1)
Supplement: Supplementary file 11 — Additional file 11: Table S5. Detection limits of elements for ICP-MS, ppb. [file 12866_2020_2012_MOESM11_ESM.docx]

Table S5. Detection limits of elements for ICP-MS, ppb.

| **Elements** | Detection limits | **Elements** | Detection limits |
| --- | --- | --- | --- |
| **Li** | 0.035 | **Cd** | 0.001 |
| **Be** | 0.0007 | **Sn** | 0.0019 |
| **B** | 1 | **Sb** | 0.003 |
| **Na** | 6.6 | **Te** | 0.006 |
| **Mg** | 0.23 | **I** | 0.067 |
| **Al** | 0.17 | **Cs** | 0.00033 |
| **Si** | 0.56 | **Ba** | 0.0029 |
| **P** | 4.5 | **La** | 0.00031 |
| **S** | 53 | **Ce** | 0.0011 |
| **Cl** | 2.9 | **Pr** | 0.0004 |
| **K** | 0.86 | **Nd** | 0.0003 |
| **Ca** | 0.97 | **Sm** | 0.0005 |
| **Ti** | 0.020 | **Eu** | 0.0002 |
| **V** | 0.0012 | **Gd** | 0.0008 |
| **Cr** | 0.014 | **Tb** | 0.0002 |
| **Mn** | 0.0087 | **Dy** | 0.0002 |
| **Fe** | 1 | **Ho** | 0.00004 |
| **Co** | 0.0014 | **Er** | 0.00005 |
| **Ni** | 0.027 | **Tm** | 0.00007 |
| **Cu** | 0.066 | **Yb** | 0.0001 |
| **Zn** | 0.079 | **Lu** | 0.0002 |
| **Ga** | 0.0009 | **Hf** | 0.0003 |
| **Ge** | 0.002 | **Ta** | 0.0002 |
| **As** | 0.0071 | **W** | 0.0017 |
| **Se** | 0.16 | **Re** | 0.0001 |
| **Br** | 0.5 | **Os** | 0.00004 |
| **Rb** | 0.0012 | **Ir** | 0.0002 |
| **Sr** | 0.0012 | **Pt** | 0.0003 |
| **Y** | 0.00019 | **Au** | 0.0005 |
| **Zr** | 0.0019 | **Hg** | 0.0045 |
| **Nb** | 0.0003 | **Tl** | 0.0002 |
| **Mo** | 0.014 | **Pb** | 0.0042 |
| **Ru** | 0.0028 | **Bi** | 0.0005 |
| **Rh** | 0.00008 | **Th** | 0.00011 |
| **Pd** | 0.0008 | **U** | 0.00034 |
| **Ag** | 0.00065 |  |  |
